# Fabrication and characterization of Ti–12Mo/xAl_2_O_3_ bio-inert composite for dental prosthetic applications

**DOI:** 10.3389/fbioe.2024.1412586

**Published:** 2024-07-15

**Authors:** Hossam. M. Yehia, Ahmed El-Tantawy, Omayma A. Elkady, Ibrahim M. Ghayad, Walid M. Daoush

**Affiliations:** ^1^ Faculty of Technology and Education, Department of Production Technology, Helwan University, Cairo, Egypt; ^2^ Central Metallurgical Research and Development Institute (CMRDI), Powder Technology Department, Helwan, Cairo, Egypt; ^3^ Central Metallurgical Research and Development Institute (CMRDI), Corrosion Control and Surface Protection Department, Helwan, Cairo, Egypt; ^4^ College of Science, Department of Chemistry, Imam Mohammad Ibn Saud Islamic University(IMSIU), Riyadh, Saudi Arabia

**Keywords:** powder metallurgy, titanium–molybdenum biomaterials, simulated artificial saliva, denture base materials, hardness, wear rate, corrosion resistance

## Abstract

**Introduction:** Titanium (Ti)-molybdenum(Mo) composites reinforced with ceramic nanoparticles have recently significant interest among researchers as a new type of bio-inert material used for dental prosthetic applications due to its biocompatibility, outstanding physical, mechanical and corrosion properties. The current work investigates the impact of alumina (Al_2_O_3_) nanoparticles on the properties of the Ti–12Mo composite, including microstructure, density, hardness, wear resistance, and electrochemical behavior.

**Methods:** Ti–12Mo/xAl_2_O_3_ nanocomposites reinforced with different Al_2_O_3_ nanoparticles content were prepared. The composition of each sample was adjusted through the mechanical milling of the elemental constituents of the sample for 24 h under an argon atmosphere. The produced nanocomposite powders were then cold-pressed at 600 MPa and sintered at different temperatures (1,350°C, 1,450°C, and 1,500°C) for 90 min. Based on density measurements using the Archimedes method, the most suitable sintering temperature was found to be 1,450°C. The morphology and chemical composition of the milled and sintered composites were analyzed using back-scattering scanning electron microscopy (SEM) and X-ray diffraction (XRD).

**Results and Discussion:** The results showed that the addition of Mo increased the Ti density from 99.11% to 99.46%, while the incorporation of 15wt% Al_2_O_3_ in the Ti–12Mo composite decreased the density to 97.28%. Furthermore, the Vickers hardness and wear behavior of the Ti–Mo composite were enhanced with the addition of up to 5 wt% Al_2_O_3_. The sample contains 5 wt% Al_2_O_3_ exhibited a Vickers hardness of 593.4 HV, compared to 320 HV for pure Ti, and demonstrated the lowest wear rate of 0.0367 mg/min, compared to 0.307 mg/min for pure Ti. Electrochemical investigations revealed that the sintered Ti–12Mo/xAl_2_O_3_ nanocomposites displayed higher corrosion resistance against a simulated artificial saliva (AS) solution than pure Ti. The concentrations of Ti, Mo, and Al ions released from the Ti–12Mo/xAl_2_O_3_ nanocomposites in the AS solution were within the safe levels. It was found from this study that; the sample of the composition Ti–12Mo/5wt%Al_2_O_3_ exhibited appropriate mechanical properties, biocompatibility, corrosion resistance against the AS solution with acceptable ion concentration released in the biological fluids. Therefore, it can be considered as a new bio-inert material for potential applications in dental prosthetics.

## Graphical Abstract

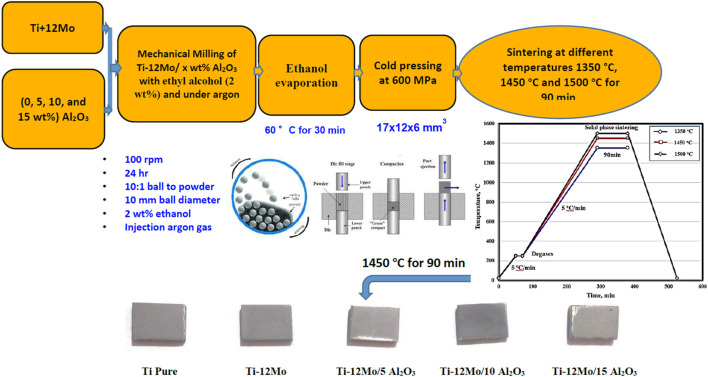

## 1 Introduction

Because of the great interest in biocompatible parts for the human body, biomaterials became an interest area for all researchers. Biomaterials include all types of materials that can be used to assist in the regeneration, repair, support, and replacement of some broken or damaged parts of human bodies. Based on the tissue response, biomaterials can be classified into three main categories, which are bio-inert, bioactive, and biodegradable materials. This classification is governed by the interaction and response of biomaterials with the host tissues. Bio-inert or bio-inactive materials are a group of materials that impregnate the body and have a minimum interaction with its tissue, e.g., stainless steel, titanium, alumina, zirconia, and polyethylene. Generally, the tissue of the human body which is in contact with the bio-inert implant might form a fibrous capsule around it. The bioactive material group enhances the interaction and bonding with the surrounding tissues. Synthetic hydroxyapatite (HAP) is an example of bioactive materials. On the other hand, tri-calcium phosphate, magnesium oxide, calcium oxide, and calcium carbonate are considered biodegradable materials (Williams, 2008; John, 2012a; John, 2012b; Liang et al., 2014; Islam et al., 2017; El-Tantawy et al., 2020; Hossam et al., 2020; Shokouh et al., 2021; Xie et al., 2021; Al-Shalawi et al., 2023; Kozłowski et al., 2023; Abd-Elaziem et al., 2024; Wei et al., 2024).

Pure elements do not have suitable strength so they need to be combined with other alloying elements to improve their physical and mechanical characteristics. The combination of more than one alloying element produces material with different properties associated with each component, and this can be achieved by different methods such as casting, extrusion, and powder metallurgy. The process of powder metallurgy is the best technique that can be used to manufacture composites with homogeneous microstructure and unique physical, mechanical, and corrosion properties (Hossam et al., 2022; Hossam et al., 2023a; Hossam et al., 2023b)**.**

Because titanium and its alloys are characterized by their low density, excellent biocompatibility, unique corrosion properties, and good mechanical performance, they were used for dental and orthopedic applications. Ti–6Al–4V, Ti–5Al–2.5Fe, and Ti–6Al–7Nb are the most commonly used composites for dental applications. It was reported from previous works that the alloy debris and the released ions in the human body fluids play an important role in the increment of the inflammation and osteolysis due to the contact with the implant parts. Because of the toxicity potential of the Ti–6Al–4V alloy, it was considered unsuitable for orthopedic applications (Xingfeng et al., 2012; K et al., 2008). Many efforts were exerted to produce elastic β-Ti alloys free from the elemental Al and V.

β-Type Ti alloys contain higher amounts of β-stabilizers such as Mo, Ta, and Zr, which are non-toxic. It has intermediate values of yield strength, modulus of elasticity, and spring back compared to stainless steel. In addition, it has lower resistance to deformation than the alpha modification. The beta phase improves the Ti alloy formability. These types of alloys possess high biocompatibility compared with other types of Ti alloys. The Ti–13Nb–13Zr and Ti–15Mo proprietary alloys are an example. Table 1 lists the different properties of the Ti base materials (Xingfeng et al., 2012; K et al., 2008; Ramakrishna et al., 2001; Chen et al., 2020; Liu et al., 2023; Lilong et al., 2017; Niinomi et al., 2001; Reham and Swain, 2015; Steinemann et al., 1980; Kawahara et al., 1963; Daoush et al., 2015; Calin et al., 2013).

**TABLE 1:** Different properties of Ti-based alloys for biomaterial applications (Xingfeng et al., 2012; K et al., 2008; Ramakrishna et al., 2001; Chen et al., 2020; Liu et al., 2023; Lilong et al., 2017; Niinomi et al., 2001; Reham and Swain, 2015; Steinemann et al., 1980; Kawahara et al., 1963; Daoush et al., 2015; Calin et al., 2013).

Comparison	*α*-Ti alloy	β-Ti alloy	(*α*–β) Ti alloy
Crystal structure	Hcp	Bcc	(*α*–β) Mixed structure dependent on alloy composition and heat treatment conditions
Eutectic temperature	Below 882°C in pure Ti	Higher than 882°C in pure Ti	
Stabilizer *α*–β	Replacement elements 1-Active elements which change the (*α*–β) transformation temperature such as Al. 2-Neutral elements which did not influence the transformation temperature such as Sn and Zr (dissolved and strengthening both *α* and β phases) Interstitial elements such as O, N, and C	Replacement elements 1-Isomorphous elements which have complete mutual solubility in the β phase, e.g., Mo, V, Nb, and Ta. 2-Eutectoid elements which were soluble in β-phase forming intermetallics with Ti with eutectoid decomposition of the beta-phase. 3-Sluggish elements which have low reactivity with Ti forming a small content of intermetallics, Fe, Cr, and Mn. 4-Active elements which form intermetallics with Ti decomposing the beta phase to alpha and intermetallics below the eutectoid temperature and can be controlled to increase the strength of the beta phase, Cu and Si. 5-Interstitial elements Hydrogen which is soluble in Ti in low and high temperatures	There are two conditions for (*α*/β) ratio: 1-The stabilizer content 2-The heat treatment conditions for enhancing of the β-phase The addition of β-stabilizer increases the alloy fabrication in both cold and hot working operations
Heat treatment	Non-heat treatable	Heat treatable	Heat treatable to varying extent
Strength	Low-medium strength	High strength up to intermediate temperature levels	Medium–high strength
Bio-compatibility	Lower bio-compatibility than the beta phase	Higher bio-compatibility than the alpha phase	Dependent on the β-phase content

Molybdenum (Mo) is biocompatible and non-allergic when its ion release content is below 8.5 μg/L (Chen et al., 2020; Liu et al., 2023). It has been reported that adding molybdenum to titanium alloys increases its corrosion resistance and strength and lowers the elastic modulus. It also acts as an effective solid solution-strengthening agent. Molybdenum insignificantly influenced superplastic deformation behavior at high temperatures due to a high fraction of the β-phase of 22%–62% (Calin et al., 2013; Daoush et al., 2015). Ti–Mo binary alloys containing from 4% to 20% Mo have considerable interest because of their simplicity, suitable cost, and their unique ability to offer a very small number of artifacts in the diagnosis by magnetic resonance imaging (MRI) compared with other β-titanium alloys (Gonzalez and Mirza-Rosca, 1999; Ho et al., 1999; Kumar and Narayanan, 2008; Disegi, 2009; Oliveira and Guastaldi, 2009; Li et al., 2010; Kandavalli et al., 2021).

Bio-ceramics such as alumina, zirconia, and HAP are used in orthopedic and denture applications (Jablokov et al., 2005; Hossam et al., 2020; Xie et al., 2021). They have great biomedical applications due to their biocompatibility with biological fluids and mechanical properties such as high stiffness and excellent wear resistance. Other unique characteristics of ceramics are high durability against corrosive chemicals and its insolubility in water, which prevent material degradation during allergic reactions and immune responses to human body fluids (Lin et al., 2002).

Alumina exhibits a low density of 3.99 g/cm^3^ and possesses remarkable characteristics such as excellent hardness, good stability, high oxidation resistance, an extremely low coefficient of friction, and favorable biocompatibility (Skinner, 1999; Chang and Billau, 2007; Al-Sanabani et al., 2014). However, its practical applications are hindered by its limited fracture toughness. To address this limitation, researchers have developed composites (Mingxian et al., 2015; Zhang et al., 2019). Among these composites, the Ti/Al_2_O_3_ system is particularly noteworthy in research studies due to its advantageous physicochemical compatibility, similar thermal expansion coefficient, and complementary mechanical properties of titanium and alumina. Ongoing research has uncovered a drawback of Ti/Al_2_O_3_ composites: a pronounced interfacial reaction between titanium and alumina occurs at elevated temperatures. This reaction generates brittle compounds such as Ti_3_Al and TiAl, which diminish the mechanical properties of the cermet at room temperature (Hanqing et al., 2016). Consequently, reducing the proportion of the brittle phase holds great significance.

Edalati et al. (2011) conducted a study that elucidated the fabrication process of titanium-based nanocomposites with varying weight percentages of Al_2_O_3_ through severe plastic deformation of the powder mixture. The results demonstrated a consistent increase in Young’s modulus and hardness values as the alumina content in the composites was augmented, albeit at the expense of reduced fracture toughness. Two other studies (Atiyah et al., 2013; Gutierrez-Gonzalez et al., 2014) focused on producing Al_2_O_3_/Ti composites using spark plasma sintering (SPS). However, Atiyah et al. (2013) encountered difficulties achieving full densification of the specimens at 1,500°C under 17 MPa. Conversely, Gutierrez-Gonzalez et al. (2014) presented mechanical property data for the alumina–titanium composite comprising 72.5 wt% Al_2_O_3_, reporting a hardness of approximately 150 GPa and a flexural strength of approximately 350 MPa. In 1970, alumina found clinical application in total hip prostheses, and it is anticipated to be utilized in cement production due to its exceptional biocompatibility, which provides enhanced stability and reduced fixation (Boutin et al., 1988). Moreover, alumina lacks bioactivity and fails to establish a direct interface with bone, thereby mitigating micro-motion (Boutin, 1971; Semlitsch et al., 1977; Harms and Mäusle, 1979; Boutin et al., 1988; Oonishi et al., 2000; Oonishi et al., 2002a; Oonishi et al., 2002b; Swan et al., 2005).

Over the past few years, the impregnation of nanoparticles has received significant research attention for decreasing the biomaterials’ corrosion rate. The process focuses on using either organic or inorganic nanoparticles on the substrate surface (Huang, 2003; Hannouche et al., 2005; Yao et al., 2005; Reda et al., 2014; Hossam and Allam, 2021). Nanoparticles also initiate selective oxidation on the surface of biomaterials, such as titania, alumina, and hydroxyapatite by forming a tenacious oxide (Boutin, 1971; Harms and Mäusle, 1979; Swan et al., 2005; Xie et al., 2021).

The fabrication of Ti matrix composites reinforced with Al_2_O_3_ has garnered significant interest among researchers globally, primarily attributed to the benefits of having a fine grain size and addressing the issue of poor adhesion between the Al_2_O_3_ particles and the Ti matrix (Cai and Zhang, 2006; Ai, 2008). Several studies (Kennedy and Karantzalis, 1999; Wua et al., 2011; Jianfeng and Ruijuan, 2013) have demonstrated that including Mo can enhance the wettability between the ceramic and metallic phases. Additionally, it serves to prevent the aggregation of ceramic particles, thereby leading to modifications in the microstructure of the fabricated composites.

The strength of the study lies in replacing V and Al alloying elements in the Ti–Al–V alloy with Mo and Al_2_O_3_ to improve the formability and coexistence of the Ti composite with the human body and reduce the Ti composite released ions in the human body. It also aims to improve the adhesion of Al_2_O_3_ with Ti using Mo. This has been achieved by adding Mo with 12 wt% and Al_2_O_3_ with different percentages of 0, 5, 1, and 15 wt% by powder metallurgy. The density, composition, microstructure, hardness, wear resistance, corrosion behavior, and ion release in artificial saliva (AS) of the fabricated Ti nanocomposites were studied.

## 2 Materials and Methods

### 2.1 Materials

For this study, high-purity Ti powder (99.8%) of particle size 45 μm purchased from Tohotec Inc., Mo powder of particle size 100 μm provided from Dop. Turkey), and Al_2_O_3_ nanoparticles of particle size 200 nm purchased from Zircar Co. Ltd. were used as starting materials to prepare the Ti nanocomposite. A mixture of 88 wt% Ti and 12 wt% Mo as a matrix composite was reinforced with 0, 5, 10, and 15 wt% Al_2_O_3_ by mechanical milling for 24 h. During the milling process, high-purity ethanol with 2 wt% was added as a controlling agent to avoid cold welding and bonding between the powder particles and the balls. The milling process was performed under an argon atmosphere to avoid any oxidation of Ti and maintain the milling process temperature. The mechanical alloying conditions were optimized at the speed of 100 rpm by using balls of 10 mm diameter. The ratio of ball to powder was adjusted to 10:1. The obtained powder was dried after the milling process at 60°C for 30 min to remove any remaining ethanol.

The prepared composite powders were compacted under 600 MPa in a rectangular die with a cross section of 17 × 12 mm^2^ using uniaxial hydraulic press and then sintered at three different temperatures of 1,350°C, 1,450°C, and 1,500°C for 90 min.

**FIGURE 1:**
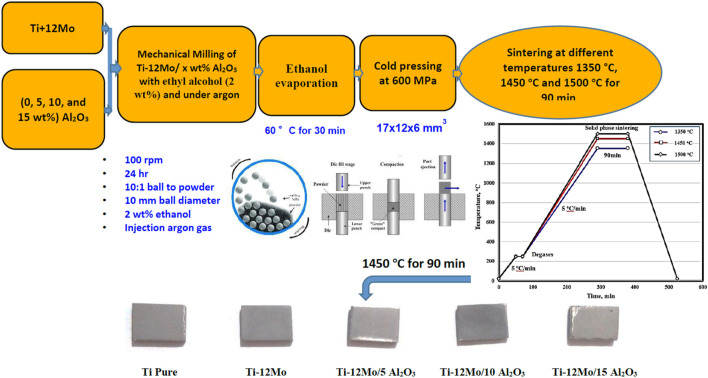
Fabrication steps of the Ti nanocomposites.

The fabrication steps of the proposed Ti nanocomposites are presented in Figure 1. To investigate the microstructure of the fabricated nanocomposites, they were grounded using SiC papers and polished with diamond paste. The metallography of the Ti, Mo, Al_2_O_3_, and Ti–12Mo/xAl_2_O_3_ powders, as well as the sintered nanocomposites, was studied using the scanning electron microscope (SEM) of model Quanta FEG250-EDAX Genesis. The composition and crystal structure of both powder and sintered nanocomposites were investigated by the X-ray diffraction (XRD) of the model D8 kristalloflex.

### 2.2 Determination of theoretical and sintered density

The theoretical density of the Ti nanocomposite was calculated by the rule of mixture according to Eq. 1 (Hossam and Allam, 2021; Hossam et al., 2023b):

$$\rho_{th.}=\rho_{1}{wt.\%}_{1}+\rho_{2}{wt.\%}_{2}+\rho_{3}{wt.\%}_{3}+\ldots ,$$ (1)

where ρ_th_ is the theoretical density, ρ_1_ is the density of the matrix element, and ρ_2_ and ρ_3_ are the density of dispersion phases.

The bulk densities of the sintered Ti samples were determined using the Archimedes method according to Eq. 2 (Hiraoka et al., 1994):

$$\rho_{Arch.}=\frac{w_{air}}{\left(w_{air}-w_{water}\right)}$$ (2)

where W_air_ and W_water_ are the weight in air and water, respectively.

### 2.3 Hardness and wear measurements

The hardness of the manufactured nanocomposites was measured at room temperature using the Vickers hardness tester of the model (Matsuzawa JAPAN). The applied load was optimized at 20 kg for 15 s. The test was repeated five times at different regions along the cross section of each specimen, and the average of the obtained values was calculated.

The wear property was measured using the pin-on-ring test machine according to the optimum conditions listed in Table 2 (Hossam et al., 2020; Elkady et al., 2021; Nour-Eldin et al., 2022). Before proceeding with the test, the specimen and ring were ultrasonically cleaned and washed several times with acetone. The weight loss was determined using an electronic digital balance of 0.0001 g sensitivity and then divided by the test time to estimate the wear rate. The average of measured wear rate values for each sample was recorded.

**TABLE 2:** Optimum wear rate test conditions.

Specimen size	12 mm × 10 mm × 6 mm
Disc material	Stainless steel (62 HR)
Load	20 N
Rotating speed	300 rpm
Wear type	Dry
Test temperature	Room temperature
Test time	30 min

### 2.4 Electrochemical and corrosion rate measurements

The corrosion property of the Ti–12Mo/x Al_2_O_3_ nanocomposites was measured at room temperature by an electrochemical working station of the model (Autolab, PGSTA30, Netherlands). The electrolyte was prepared from AS. The AS chemical composition is listed in Table 3 (Huang, 2003; REDA et al., 2014). The cross-section area of each tested sample was 0.204 cm^2^. The samples were grounded with SiC papers to prepare their surfaces for the test. The sample was the working electrode. The counter platinum electrode and reference saturated calomel electrode (SCE) were used to close and complete the electric circuit.

**TABLE 3:** Chemical composition of artificial saliva simulated fluid.

Component	Concentration (g/L)
NaCl	0.4
KCl	0.4
CaCl_2_.2H_2_O	0.795
NaH_2_PO_4_.H_2_O	0.690
KSCN	0.30
Na_2_S.9H_2_O	0.005
Urea	1
pH	Slightly acidic (∼4)

The open-circuit potential (OCP) was recorded in the AS solution for 30 min. Potentiodynamic polarization was measured up to 3.0 V with a scan rate of 2 mVs^−1^. The corrosion rates were evaluated by applying the Tafel method based on the polarization plots.

To evaluate the ion release of the fabricated samples, an immersion test was performed in the prepared AS solution at a pH of four according to the standard ASTM-G31-72 (Reda et al., 2014). The specimens were immersed in 90 mL for 14 days at 37°C. The concentration of the released ions of each composite was determined by inductively coupled plasma (ICP) atomic emission spectrometry (Perkin Elmer Inc. Optima 2000 DV).

## 3 Results and discussion

### 3.1 Powders’ particle size, shape, and morphology

**FIGURE 2:**
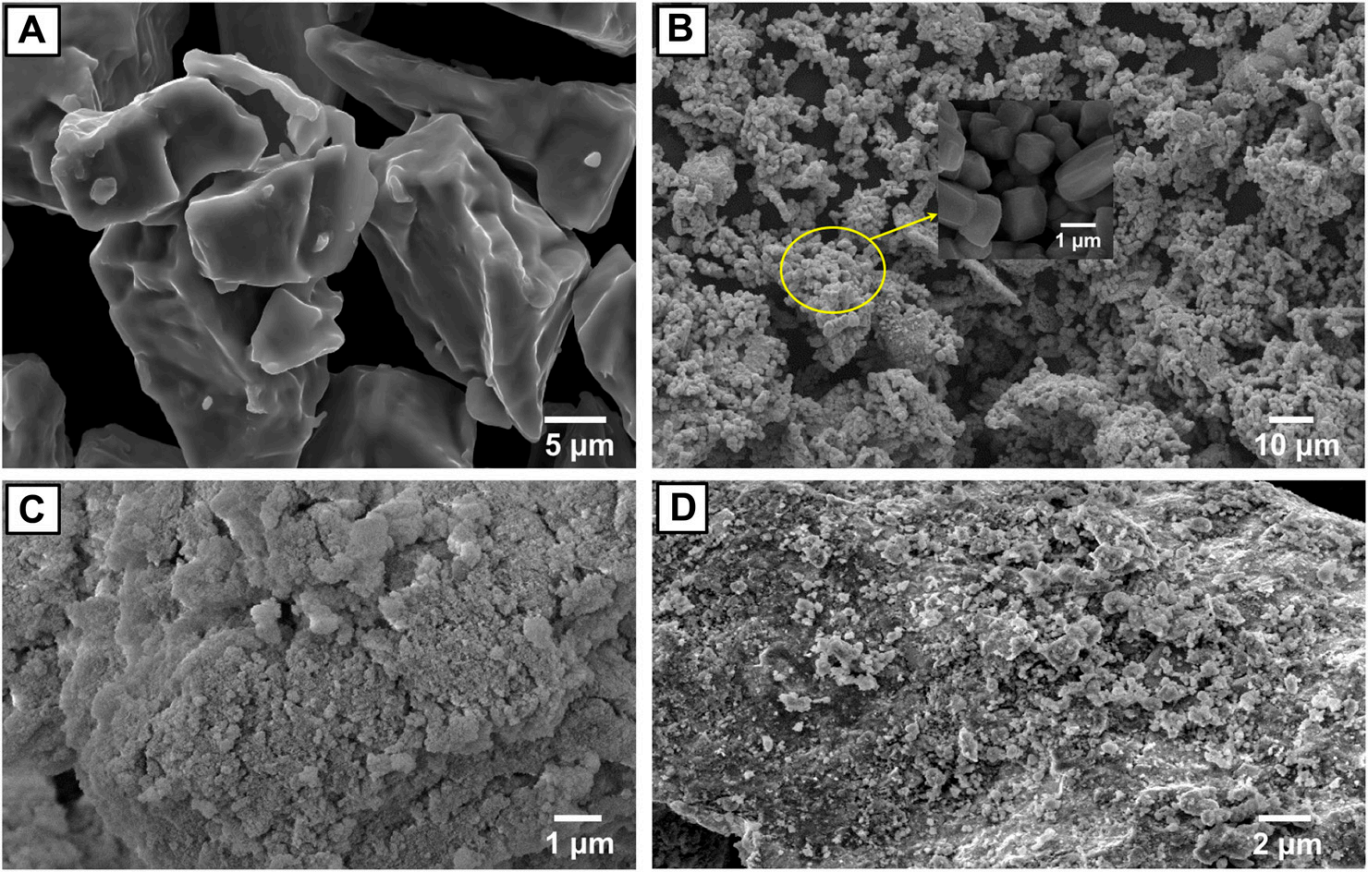
SEM images of **(A)** Ti, **(B)** Mo, **(C)** Al_2_O_3_ nanoparticles, and **(D)** Ti–12Mo/15 wt% Al_2_O_3_ nanocomposite mixture.

Figure 2A–D represent the SEM images of the Ti, Mo, and Al_2_O_3_ powders, as well as the milled Ti–12Mo/15 wt% Al_2_O_3_ nanocomposite. The Ti particles are irregular and have sharp edges. The Mo particles have hexagonal and cubic shapes. The Al_2_O_3_ powder is very fine and in the nano-size. The elements have gray and dark gray colors. Due to milling the Ti–12Mo/15 wt% Al_2_O_3_ nanocomposite powder for 24 h, the constituent reduced to the nanoscale, as shown in Figure 2D.

### 3.2 X-ray diffraction and phase identification

**FIGURE 3:**
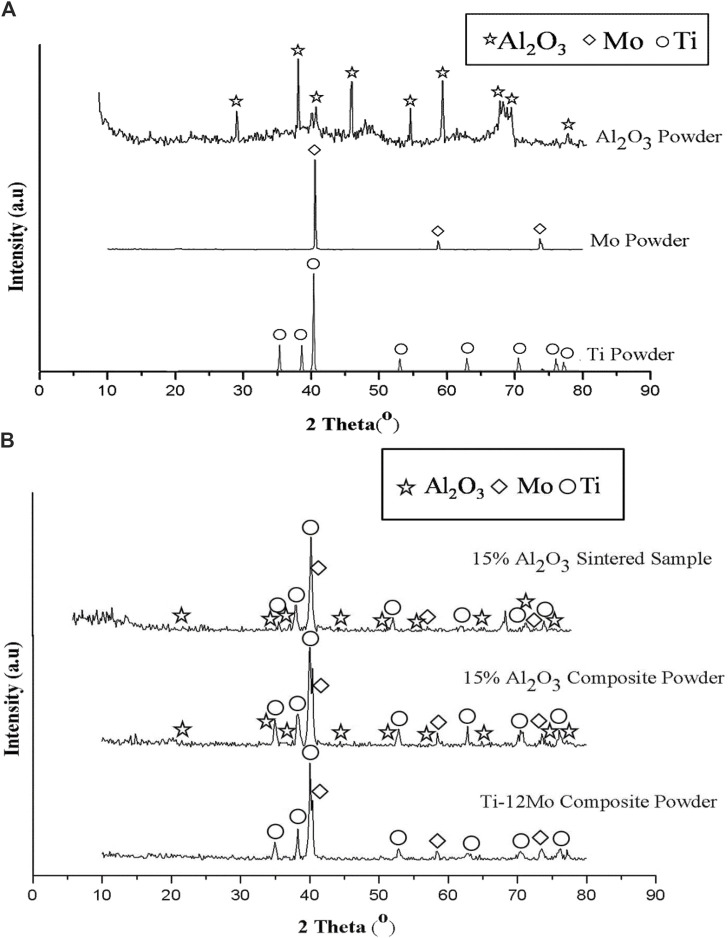
XRD patterns of **(A)** Ti, Mo, and Al_2_O_3_ powders, and **(B)** Ti–12Mo, Ti–12Mo/15 wt% Al_2_O_3_ nanocomposites mixture, and Ti–12Mo/15 wt% Al_2_O_3_ sintered nanocomposite.

The chemical composition and crystal structure of the starting materials Ti, Mo, and Al_2_O_3_ nanoparticles were investigated using the XRD analysis. As shown in Figure 3A, there are no any foreign phases appeared rather than Ti, Mo, and Al_2_O_3_, which confirmed their high purity. The analysis emphasizes that the Ti metal has HCP, Mo has BCC, and Al_2_O_3_ has a rhombohedral crystal structure.

Figure 3B shows the XRD of the Ti–12Mo powder, Ti–12Mo/15 wt% Al_2_O_3_ powder, and the Ti–12Mo/15 wt% Al_2_O_3_ sintered sample. No foreign peaks were detected after both milling and sintering processes. The peak broad can interpret the particle size, where the wider the peak, the smaller the particle size (Reda et al., 2014; Yehia et al., 2022). By comparing the width of pure element peaks and the nanocomposites before and after sintering, it is noted that the peak broadening after milling and sintering is wider due to the decreasing particle size after the milling process for 24 h.

### 3.3 Microstructure investigation

**FIGURE 4:**
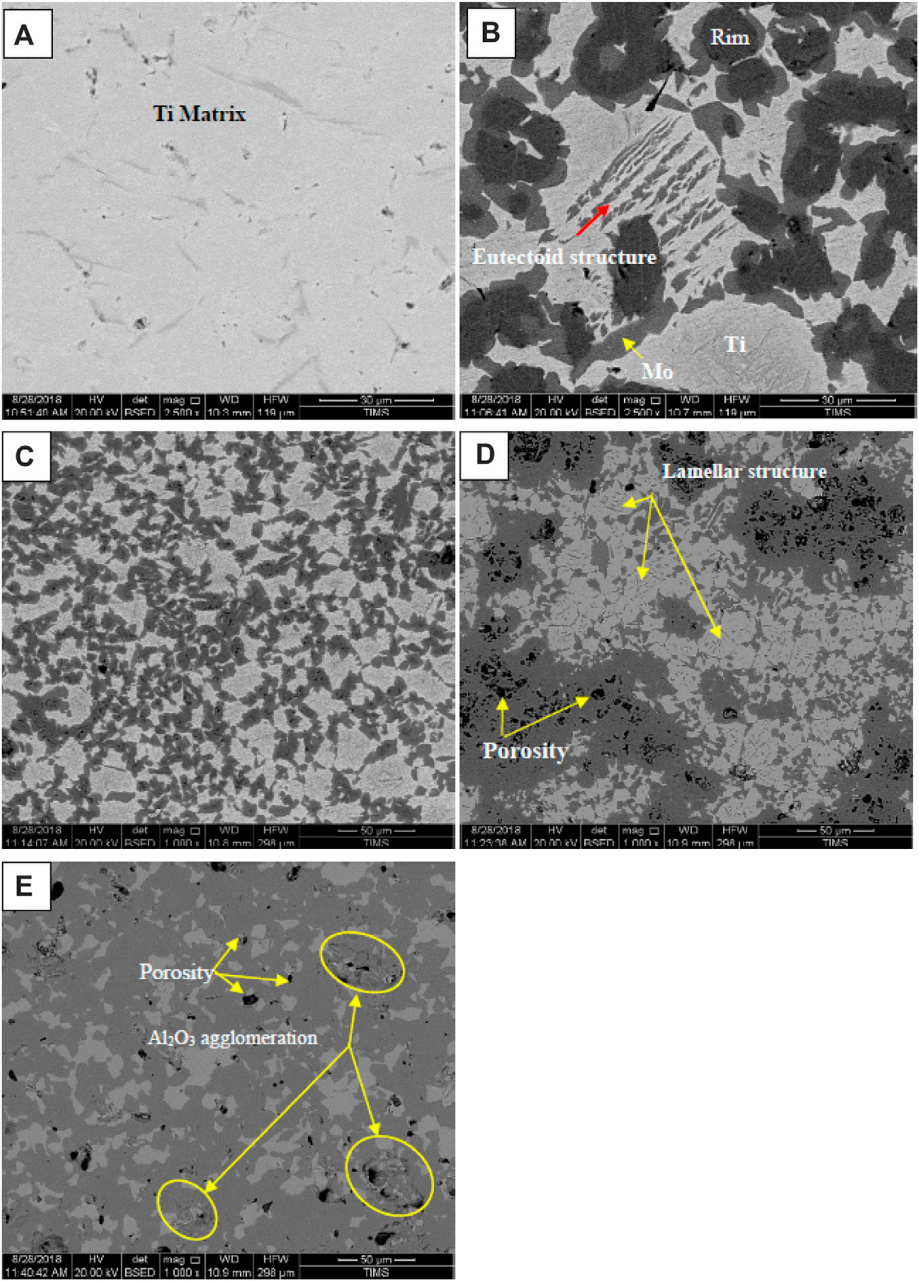
SEM images of **(A)** Ti pure, **(B)** Ti–12Mo, **(C)** Ti–12Mo/5 wt% Al_2_O_3_, **(D)** Ti–12Mo/10 wt% Al_2_O_3_, and **(E)** Ti–12Mo/15 wt% Al_2_O_3_ nanocomposites sintered at 1,450°C for 90 min.

Figure 4 shows the SEM images of the pure titanium, Ti–12Mo, and Ti–12Mo/x Al_2_O_3_ nanocomposites with different Al_2_O_3_ nanoparticle contents (*x* = 5, 10, and 15 wt%). It can be observed from the microstructure presented in image (a) that pure Ti has (α) phase, while the microstructure of the Ti–12Mo nanocomposite in image (b) exhibits α (Ti) and γ (Mo) phases. Some regions of the Ti–12Mo microstructure present the eutectoid structure in the form of α and γ layers (pearlite phase). As presented in previous reports of the Ti–Mo binary phase diagram system (Yehia et al., 2023), the eutectoid structure of Ti–Mo can be formed in the composition of 79 Ti to 21 Mo by wt% at 695°C. A hypo-eutectoid is expected to be established because the weight percentage of Mo is less than 21 Wt%. As shown in image **(b)**, a typical interface between the Ti and Mo particles was formed. In addition, homogeneous dispersion of the Mo with the Ti matrix was achieved due to the long mechanical milling time. A core/rim was observed due to the partial diffusion in the interface between Ti and Mo after polishing. By reinforcing the Ti–12Mo nanocomposite with the Al_2_O_3_ nanoparticles, the lamellar microstructure disappeared. In addition, the formation of pores and agglomeration of Al_2_O_3_ nanoparticles are observed in the Ti–12Mo/10–15 wt% Al_2_O_3_ nanocomposite. The pore formation may be due to the limited wettability between the Al_2_O_3_ nanoparticles and the matrix (Chen et al., 2021; El-Kady et al., 2022). The addition of nano-Al_2_O_3_ with 5 wt% affects the particle size of Mo, as shown in **image c**. The Mo particles became more fine and distributed with excellent meaner.

**FIGURE 5:**
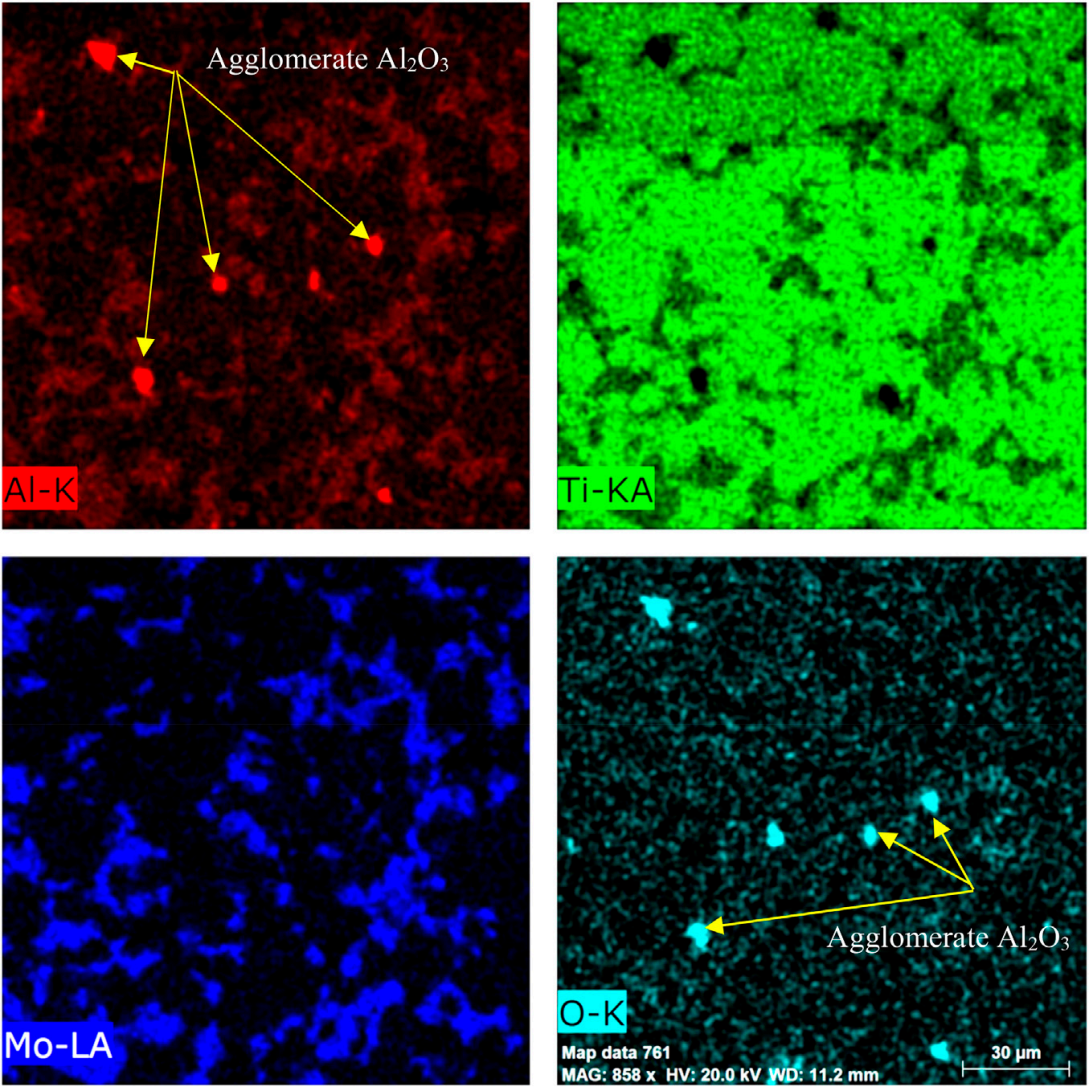
SEM mapping of the Ti–12Mo/10 wt% Al_2_O_3_ fabricated nanocomposite.

Because the dispersed phase Al_2_O_3_ was in the form of nanoparticles and was not detected by SEM, the elemental distribution by the mapping was performed for the Ti–12Mo/10 wt% Al_2_O_3_ specimen, as shown in Figure 5. Regardless of the homogeneous dispersion of Mo, some agglomerations of the Al_2_O_3_ nanoparticles were detected.

### 3.4 Density evaluation

**FIGURE 6:**
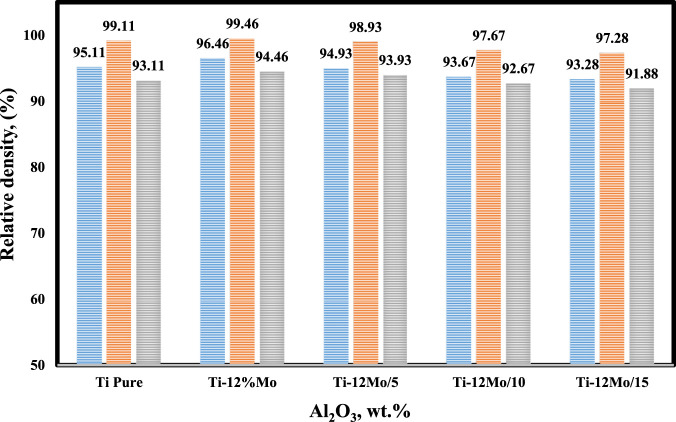
Relative density of the Ti nanocomposites at different sintering temperatures.

Figure 6 displays the effect of sintering temperature and the addition of nano-Al_2_O_3_ content up to 15 wt% on the relative density of the prepared Ti–Mo nanocomposite. It is clear that the highest relative densities among the fabricated samples were achieved at 1,450°C. The relative density of the titanium matrix was improved due to reinforcing it with 12 wt %Mo. Ti–12Mo recorded 99.46% compared with 99.11% for pure Ti. This improvement may be due to the higher density of Mo, which is 10.22 g/cm^3^ compared with 4.506 g/cm^3^ for Ti. In addition, the good adhesion and distribution of Mo with Ti are shown in **image 4b**. Previous works (Ning et al., 2003; Zhou et al., 2008; Wang et al., 2010; Kiviö et al., 2014) revealed that the addition of Mo could improve the wettability between reinforcement and metal matrix phases and prevent the agglomeration of ceramic particles. Mo had a great influence on the density, morphology, and mechanical properties. By the addition of Mo into Ti, a new phase is formed on the rim of Ti grains. The new phase aids to improve the wettability of the liquid phase, refine the grains of the hard phase, and decrease the porosity.

The Ti–12Mo nanocomposite density decreased gradually by increasing the Al_2_O_3_ content up to 15 wt%. This reduction may be due to several reasons that can be summarized as follows: the low density of Al_2_O_3_ (3.95 g/cm^3^), the agglomeration of nano-Al_2_O_3_, especially at high percentages of 10 and 15 wt%, and the poor wettability between the Al_2_O_3_ particles as a ceramic material and the Ti–Mo metal matrix, which encourage the formation of pores.

### 3.5 Hardness

**FIGURE 7:**
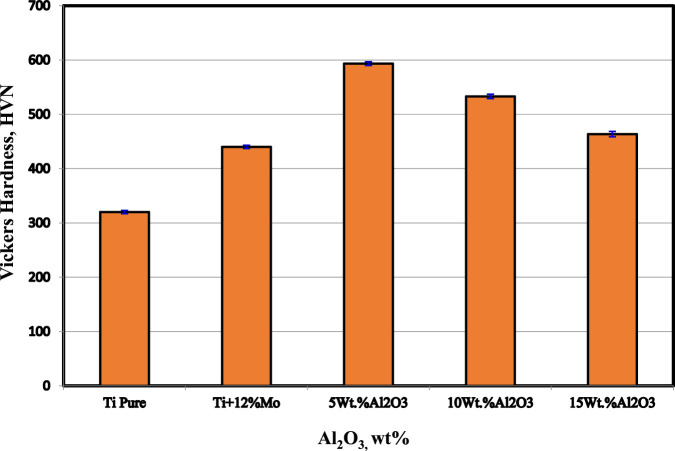
Vickers hardness of sintered nanocomposites at 1,450°C.

The hardness values of the Ti nanocomposites sintered at 1,450°C are shown in Figure 7. The results show that the hardness of pure Ti was improved by the addition of 12 wt% Mo and Al_2_O_3_ up to 5 wt%. The addition of 12 wt% Mo increases the hardness from 320 HV to 440 HV with 37.5%. On the other hand, the addition of 5 wt% Al_2_O_3_ increases the hardness from 440 HV to 594 HV with a percentage of 35%. It is expected that not only the high hardness of both Mo and Al_2_O_3_ is the main factor that participates in improving Ti hardness but also the formation of the diffused core/rim at the interface between Mo and Ti and the homogenous dispersion of the Mo and Al_2_O_3_ nanoparticles, as shown in the microstructure **images b** and **c**, have a great effect. Kiviö et al. (2014) studied the effect of Mo on the wettability of Fe/TiC alloy. They reported that by the addition of 3.7 and 5.2 wt% Mo to Fe, a decrease in the wetting angle of Fe–TiC to approximately 20° was achieved. Due to increasing the interfacial bonding strength, the mechanical properties of the TiC–Fe (Mo) composite were increased. A refinement in the particle sizes after adding 5 wt% Al_2_O_3_ was noted, confirming the increment in the hardness according to the hall pitch equation (Yehia et al., 2023). Jianfeng and Ruijuan (2013) successfully fabricated the (Ti, Mo)_2_A1C/10 wt% Al_2_O_3_ *in situ* composite from powder mixtures of Ti, Al, TiC, and MoO_3_ by reactive hot pressing sintering. The Vickers hardness of the (Ti, Mo)_2_ A1C/10 wt% Al_2_O_3_ *in situ* composite was improved by 25% compared to the single-phase Ti_2_AlC.

The presence of Al_2_O_3_ nanoparticles on the grain boundaries of the binary Ti–Mo particles may decrease the dislocation movements and consequently increase the hardness (Chen et al., 2021; Hossam and Allam, 2021; El-Kady et al., 2022). Regardless of the decrease in hardness of the Ti–12Mo/10 and 15 wt% Al_2_O_3_ nanocomposites, they are still greater than the hardness of the Ti–Mo composite. The agglomeration of Al_2_O_3_ nanoparticles and the formation pores detected from the microstructure are the main factors that affect the reduction in hardness after 5 wt% Al_2_O_3_. Moreover, the poor wettability between Al_2_O_3_ and Ti–12Mo matrix deteriorates the properties by increasing the Al_2_O_3_ nanoparticles higher than 5 wt%.

### 3.6 Wear behavior

**FIGURE 8:**
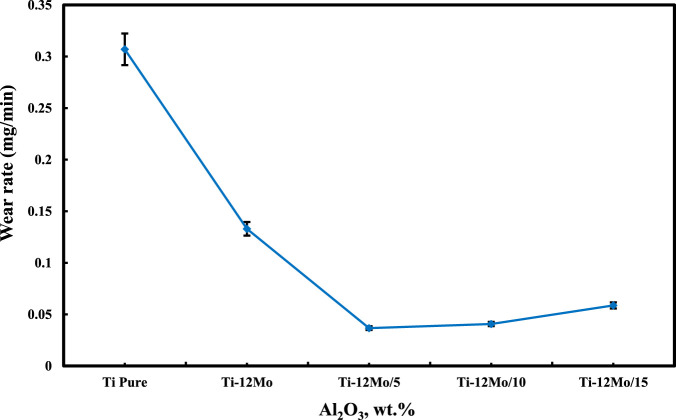
Wear rate of the Ti–12Mo/xAl_2_O_3_ nanocomposites.

The wear rate of the Ti–12Mo/xAl_2_O_3_ nanocomposites under a 20 N applied load and a sliding speed of 300 rpm for 30 min is shown in Figure 8. It is evident from the results that the wear rate of pure Ti decreased as a result of reinforcing it with Mo and different weight percentages of Al_2_O_3_ nanoparticles. Among the samples, the Ti–12Mo/5 wt% Al_2_O_3_ sample exhibits the lowest wear rate.

The decrease in the wear rate of the Ti–12Mo/5 Wt% Al_2_O_3_ nanocomposites may be attributed to the high hardness values of Mo and Al_2_O_3_ nanoparticles and the microstructure refinement. Additionally, the homogeneous distribution of Al_2_O_3_ nanoparticles enhances oxide dispersion strengthening (ODS) by reducing dislocation movements. The slight increase in the wear rate of Ti–12Mo/Al_2_O_3_ nanocomposites with the Al_2_O_3_ content higher than 5% may be due to the agglomeration of particles, which decreases the bonding strength between particles and consequently increases the wear rate (Huang, 2003; REDA et al., 2014).

In a separate study, the wear behavior of Ti–6Al–4V composites reinforced with Al, TiN, Ni60, and Si powders and coated with TiN, TiB, Ti_5_Si_3_, and Al_3_Ti, was investigated. Tests were conducted at different temperatures (25, 350, and 700°C) and loads (3, 6, and 9 N). The coated samples exhibited lower wear rates than the Ti–6Al–4V alloy (Chen et al., 2021). Another study explored the wear performance of Ti–6Al–4V composites containing TiN as a reinforcement using SPS. The addition of TiN decreased the Ti density while increasing its micro-hardness. The Ti/TiN worn surface demonstrated improved resistance to abrasive wear (Falodun et al., 2019).

### 3.7 Corrosion behavior of the Ti–12Mo/Al_2_O_3_ nanocomposite

#### 3.7.1 Electrochemical corrosion behavior

**FIGURE 9:**
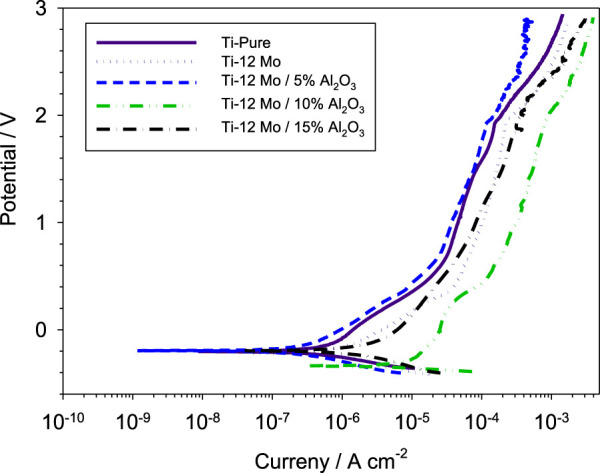
Potentiodynamic polarization curves of the investigated Ti–12Mo/xAl_2_O_3_ nanocomposites in contact with the AS solution.

Figure 9 shows the polarization behavior of the fabricated specimens against a simulated AS solution. The Ti–12Mo/xAl_2_O_3_ nanocomposites exhibit polarization behaviors similar to the pure Ti with broad passivation regions. The corrosion parameters of all nanocomposites are shown in Table 4. As shown, the 5wt% Al_2_O_3_ nanocomposite exhibited the lowest potential of 0.195 V and lowest current density of 1.123 × 10^−6^ A/cm^2^. The same sample recorded the lowest corrosion rate of 8.572 × 10^−3^ mm/year compared with the other nanocomposites. This can be due to the impregnation of the appropriate content of the Al_2_O_3_ nanoparticles. Alumina acts as a ceramic material as it resists the corrosion due to its high chemical stability. Decreasing the corrosion resistance of 10 and 15 wt% Al_2_O_3_ may be due to the presence of agglomerations and, consequently, pores that facilitate the absorption of the AS solution inside the pores of the sintered samples; consequently, the corrosion rate is increased.

**TABLE 4:** Corrosion parameters of the electrochemical measurements of the tested nanocomposites.

Material	E _corr_ (V)	I_corr_ (A/cm^2^)	B_c_ (V/dec)	B_a_ (V/dec)	Corrosion rate (mm/year)
Pure Ti	0.204	1.842 × 10^−6^	0.108	0.448	1.605 × 10^−2^
Ti–12Mo	0.212	3.416 × 10^−6^	0.375	0.148	2.577 × 10^−2^
Ti–12Mo/5% Al_2_O_3_	0.195	1.123 × 10^−6^	0.15	0.406	8.572 × 10^−3^
Ti–12Mo/10% Al_2_O_3_	0.338	4.984 × 10^−6^	0.025	0.323	4.138 × 10^−2^
Ti–12Mo/15% Al_2_O_3_	0.196	2.856 × 10^−6^	0.159	0.745	2.397 × 10^−2^

**FIGURE 10:**
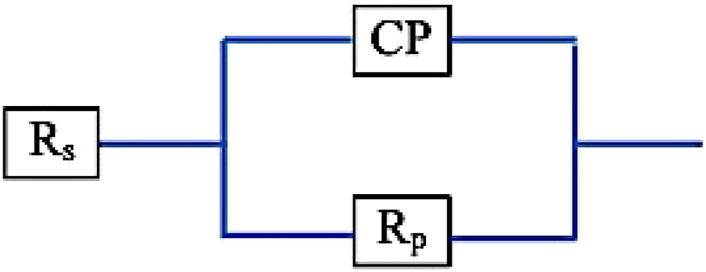
Schematic diagram of the equivalent circuit for curve-fitting results of EIS.

**FIGURE 11:**
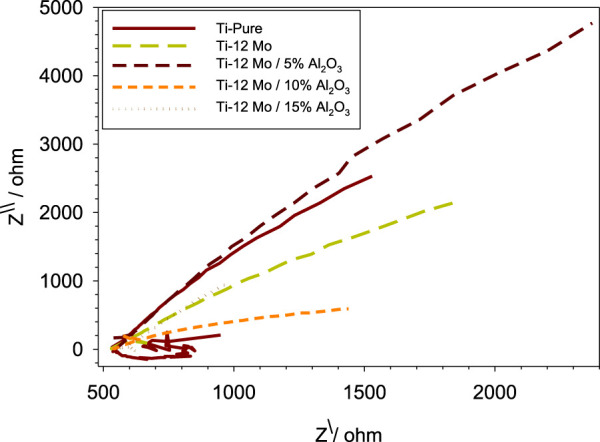
Nyquist plots of the fabricated nanocomposites in the AS solution.

**FIGURE 12:**
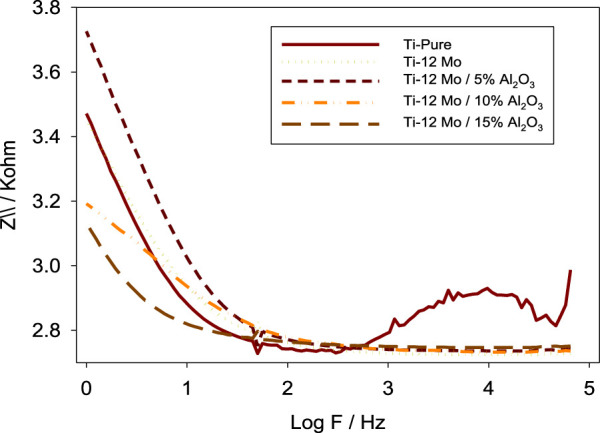
Bode plots of pure titanium and Ti–12Mo/xAl_2_O_3_ nanocomposites in the AS solution.

The electrochemical impedance spectroscopy (EIS) of each specimen surface was performed in the range of 1 Hz–65 kHz at the amplitude of 10 mV in the open-circuit potential, as shown in Figure 10. Figure 11 shows Nyquist plots of the prepared Ti–12Mo/Al_2_O_3_ nanocomposites. The Nyquist plot shows the measuring frequency region of one-semicircle that is due to the formation of a complete film of passive barrier on the surface of the nanocomposite sample (Daoush et al., 2016; Sayed et al., 2017; Elsayed et al., 2020). The obtained parameters are directly computed by the mean of software of the EIS testing device, as listed in Table 5. It was revealed that the polarization resistance reaches the highest value of 18.4015 kΩ in the Ti–12Mo/5% Al_2_O_3_ nanocomposite, indicating an improvement in the corrosion resistance. Generally, it was also observed that the Ti–12Mo/Al_2_O_3_ nanocomposites exhibit better corrosion resistance than either pure Ti or Ti–12Mo metallic composite. Figure 12 shows the Bode plots of the investigated samples which represents the same behavior observed in the Nyquist plot. Both plots show high-frequency inductive loops of pure Ti and Ti–12Mo/15% Al_2_O_3_ nanocomposites which may be attributed to the adsorption process of the species on the surface of the samples during the test in the AS solution.

**TABLE 5:** Corrosion parameters of pure Ti and Ti–12Mo/xAl_2_O_3_ nanocomposites obtained from impedance measurements in the AS solution.

Material	Rs (Ohm)	Rp (Ohm)	CPE (F)	N
Pure Ti	5.89157 × 10^2^	1.52927 × 10^4^	6.64787 × 10^−5^	0.88466
Ti–12Mo	5.28009 × 10^2^	1.58883 × 10^4^	9.25407 × 10^−5^	0.74231
Ti–12Mo/5% Al_2_O_3_	5.45862 × 10^2^	4.72131 × 10^4^	4.07435 × 10^−5^	0.83389
Ti–12Mo/10% Al_2_O_3_	5.33432 × 10^2^	2.78589 × 10^3^	2.13388 × 10^−4^	0.56137
Ti–12Mo/15% Al_2_O_3_	5.567 × 10^2^	4.37519 × 10^4^	2.43481 × 10^−4^	0.75286

#### 3.7.2 Released ions in simulated artificial saliva

**FIGURE 13:**
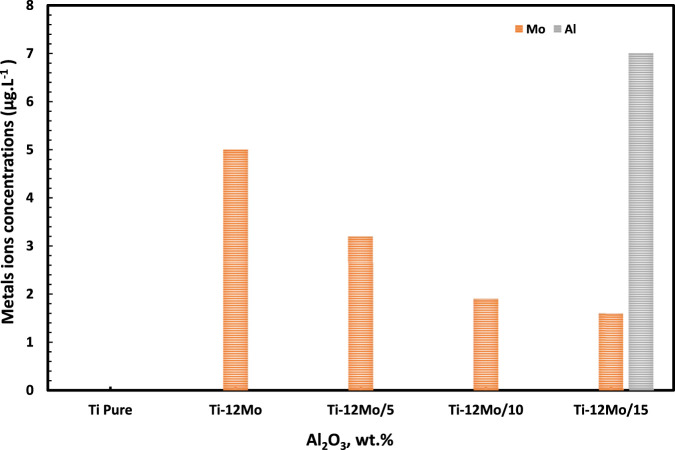
Ti, Mo, and Al ion concentrations in the AS of the Ti and Ti–12Mo/xAl_2_O_3_ nanocomposites after immersion for 14 days.

Figure 13 displays the different concentrations of Ti, Mo, and Al ions which are released from pure Ti and Ti–12Mo/Al_2_O_3_ nanocomposites after immersion in simulated AS for 14 days. It was observed from the results that the Ti ions were not detected for all tested samples. On the other hand, the Mo ion concentration of the Ti–Mo nanocomposite shows its maximum value of 5.0 parts per billion (ppb). However, in case of the Ti–12Mo/xAl_2_O_3_ composites, a significant decrease in the Mo ion concentration was observed. The Ti–12Mo/5 and 10Wt% Al_2_O_3_ nanocomposites did not release any Al ions in the AS solution. However, increasing the Al_2_O_3_ nanoparticle content to 15% in the Ti–12Mo/xAl_2_O_3_ nanocomposite 15% results in the increase of Al ions up to 7.0 ppb. It is now clear that Ti–12Mo/5 Wt% Al_2_O_3_ and Ti–12Mo/10 wt% Al_2_O_3_ show the minimum released ion concentrations in the AS solution. This may be due to the formation of the good bonding between Ti, Mo, and Al_2_O_3_ nanoparticles by the milling, compaction, and sintering process, which enhances high chemical stability of the samples. It also revealed that the concentrations of the Ti, Mo, and Al ions released from all the investigated Ti–12Mo/xAl_2_O_3_ nanocomposite samples in the simulated AS are considerably safe and have low toxicity (Vasilescu et al., 2012; REDA et al., 2014; Xu et al., 2018).

## 4 Conclusion

To enhance the mechanical properties and reduce the corrosion rate of Ti, it was reinforced with 12 wt% Mo and varying amounts of alumina nanoparticles (5, 10, and 15 wt%). The sintering process was performed at different temperatures (1,350°C, 1,450°C, and 1,500°C) to determine the optimal conditions. The Ti composites achieved the highest relative density when sintered at 1,450°C for 90 min. No new intermetallic or foreign phases were detected before or after sintering. Scanning electron microscopy (SEM) analysis of the Ti–12Mo/xAl_2_O_3_ nanocomposite revealed good dispersion of both Mo and Al_2_O_3_ nanoparticles up to 5 wt%. However, porosities were observed at higher percentages of Al_2_O_3_.

The addition of 5 wt% Al_2_O_3_ resulted in a significant increase in hardness, with a hardness value of 593.4 HV, representing an 85.4% increment compared to pure Ti (320 HV). Although the hardness decreased at 10% and 15% alumina, it still surpassed that of pure titanium. The wear resistance of Ti was improved with the incorporation of Mo and Al_2_O_3_. The addition of 5 wt% Al_2_O_3_ led to the lowest wear rate of 0.0367 mg/min, which corresponded to an 88% reduction compared to pure Ti. The corrosion rate was also reduced significantly in the 5 wt% Al_2_O_3_ sample, reaching the lowest value of 8.572 × 10^−3^ mm/year. This improvement was attributed to the absence of porosity, good distribution, and adhesion of Al_2_O_3_ nanoparticles within the Ti–12Mo matrix. Furthermore, increasing the Al_2_O_3_ content resulted in decreased ion concentration due to its high chemical stability.

In summary, the Ti–12Mo/5 wt% Al_2_O_3_ nanocomposite exhibited excellent mechanical properties, appropriate corrosion resistance against the AS solution, and acceptable ion concentration. Therefore, it is considered a promising material for potential applications in dental prosthetics.

## Data Availability

The raw data supporting the conclusion of this article will be made available by the authors, without undue reservation.
